# Supplementary material for: Root bacterial endophytes alter plant phenotype, but not physiology
Source: PeerJ. 2016 Nov 1;4:e2606. doi: 10.7717/peerj.2606 (PMC5101591; doi:10.7717/peerj.2606)
Supplement: Supplemental Information 1 — Physiology traits of Populus trichocarpa that were not inoculated with bacteria (no microbe control) (n = 8) (white) or were inoculated with Pseudomonas GM30 (n = 7), Pseudomonas GM41 (n = 8), or Burkholderia BT03 (n = 7). Across all treatments there were no significant differences in: a) Plant chlorophyll content (SPAD), b) ΦCO2 (expressed as the slope of carboxylase activity across different light levels), and c) carboxylase activity under maximum light level and CO2 concentration (Amax). [file peerj-04-2606-s001.docx]

**Supplemental material**

Supplemental Table 1: Linear mixed models results for the response of plant trait expression in response to microbial additions. Models were constructed with initial growth measurements as co-variety when appropriate, bacterial treatment as a fixed factor and experimental run as random factor. To test for significance of various factors, we constructed models with and without fixed factors and covariates and compared using likelihood ratio test. Plant traits compared included: shoot biomass, log root biomass, whole plant biomass, chlorophyll content, number of new leaves added, root area growth rate, root length growth rate, stem growth rate, specific root length, specific leaf area, leaf growth rate, log average leaf size, change in leaf size, log quantum yield of CO_2_ fixation (Φ), maximum rate leaves were able to fix carbon (A_max_), and maximum rate of photosynthesis in saturating light under ambient CO_2_ (A_sat_).

| **Trait** | **Fixed effect** | **𝜒2 or f** | **P value** | **Estimate** | **SE** | **t** | **Random effect** | **Variance** | **SD** |
| --- | --- | --- | --- | --- | --- | --- | --- | --- | --- |
| Dry shoot biomass (leaves + stem) (mg) | Bacterial Trt | 1.135 | 0.567 |  |  |  |  |  |  |
|  | Init. Leaf SA | 5.218 | **0.022** |  |  |  |  |  |  |
|  | Bac + In. L. SA | 5.038 | **0.025** |  |  |  |  |  |  |
|  |  |  |  |  |  |  |  |  |  |
|  | Intercept |  |  | 32.57 | 18.66 | 1.745 | Exp. Run | 0 | 0 |
|  | GM30 |  |  | 54.03 | 21.1 | **2.56** | Residuals | 1661 | 40.75 |
|  | GM41 |  |  | 22.71 | 20.38 | 1.114 |  |  |  |
|  | BT03 |  |  | 33.69 | 21.17 | 1.599 |  |  |  |
|  | Init. Leaf SA |  |  | 5.963 | 2.349 | **2.538** |  |  |  |
|  |  |  |  |  |  |  |  |  |  |
| Leaf dry weight (mg) | Bacterial Trt | 0.152 | 0.697 |  |  |  |  |  |  |
|  | Init. Leaf SA | 0.321 | 0.571 |  |  |  |  |  |  |
|  | Bac + In. L. SA | 6.202 | **0.045** |  |  |  |  |  |  |
|  |  |  |  |  |  |  |  |  |  |
|  | Intercept |  |  | 57.05 | 17.19 | **3.21** | Exp. Run | 338.1 | 18.39 |
|  | GM30 |  |  | 49.42 | 20.35 | **2.43** | Residuals | 1543.0 | 39.28 |
|  | GM41 |  |  | 19.13 | 19.64 | 0.97 |  |  |  |
|  | BT03 |  |  | 34.61 | 20.35 | 1.70 |  |  |  |
|  | Init. Leaf SA |  |  | 5.963 | 2.349 | **2.538** |  |  |  |
|  |  |  |  |  |  |  |  |  |  |
| Log root biomass (mg) | Bacterial Trt | 0 | 1 |  |  |  |  |  |  |
|  | Init. Root SA | 4.656 | **0.031** |  |  |  |  |  |  |
|  | Bac + In. R SA | 5.479 | **0.019** |  |  |  |  |  |  |
|  |  |  |  |  |  |  |  |  |  |
|  | Intercept |  |  | 2.824 | 0.201 | 14.06 | Exp. Run | 0 | 0 |
|  | GM30 |  |  | 0.347 | 0.272 | 1.273 | Residuals | 0.275 | 0.525 |
|  | GM41 |  |  | 0.183 | 0.263 | 0.696 |  |  |  |
|  | BT03 |  |  | 0.32 | 0.274 | 1.167 |  |  |  |
|  | Init. Root SA |  |  | 0.037 | 0.015 | 2.452 |  |  |  |
|  |  |  |  |  |  |  |  |  |  |
| Whole biomass (mg) | Bacterial Trt | 3.266 | 0.195 |  |  |  |  |  |  |
|  | Init. Leaf SA | 1.201 | 0.271 |  |  |  |  |  |  |
|  | Bac + In. L. SA | 1.707 | 0.191 |  |  |  |  |  |  |
|  |  |  |  |  |  |  |  |  |  |
|  | Intercept |  |  | 90.87 | 21.43 | 4.24 | Exp. Run | 241.9 | 15.55 |
|  | GM30 |  |  | 59.1 | 28.06 | 2.107 | Residuals | 2934.5 | 54.17 |
|  | GM41 |  |  | 22.74 | 27.09 | 0.84 |  |  |  |
|  | BT03 |  |  | 41.6 | 28.06 | 1.483 |  |  |  |
|  | Initial Leaf SA |  |  | -0.038 | 0.043 | -0.898 |  |  |  |
|  |  |  |  |  |  |  |  |  |  |
| Chlorophyll content (SPAD) | Bacterial Trt | 2.148 | 0.542 |  |  |  |  |  |  |
|  |  |  |  |  |  |  |  |  |  |
|  | Intercept |  |  | 26.075 | 2.018 | 12.92 | Exp. Run | 5.173 | 2.274 |
|  | GM30 |  |  | 1.102 | 2.178 | 0.466 | Residuals | 17.67 | 4.204 |
|  | GM41 |  |  | 1.913 | 2.102 | 0.91 |  |  |  |
|  | BT03 |  |  | -1.028 | 2.178 | -0.047 |  |  |  |
|  |  |  |  |  |  |  |  |  |  |
| Change in Leaf number | Bacterial Trt | 0 | 1 |  |  |  |  |  |  |
|  | Init. Leaf num | 18.656 | **1.6E-05** |  |  |  |  |  |  |
|  | Bac +In. Leaf # | 23.396 | **1.3E-06** |  |  |  |  |  |  |
|  |  |  |  |  |  |  |  |  |  |
|  | Intercept |  |  | 9.139 | 1.788 | 5.24 | Exp. Run | 2.433 | 1.56 |
|  | GM30 |  |  | 3.212 | 1.036 | **3.10** | Residuals | 3.997 | 1.99 |
|  | GM41 |  |  | 0.853 | 1.004 | 0.85 |  |  |  |
|  | BT03 |  |  | 1.336 | 1.049 | 1.30 |  |  |  |
|  | Init. leaf num. |  |  | -1.390 | 0.243 | **-5.73** |  |  |  |
|  |  |  |  |  |  |  |  |  |  |
| Root area growth rate (cm^2^d^-1^) | Bacterial Trt | 13.861 | **0.003** |  |  |  |  |  |  |
|  |  |  |  |  |  |  |  |  |  |
|  | Intercept |  |  | 0.126 | 0.041 | 3.039 | Exp. Run | 1.066 | 1.033 |
|  | GM30 |  |  | 0.233 | 0.06 | **3.844** | Residuals | 1.18 | 1.086 |
|  | GM41 |  |  | 0.094 | 0.058 | 1.611 |  |  |  |
|  | BT03 |  |  | 0.071 | 0.06 | 1.177 |  |  |  |
|  |  |  |  |  |  |  |  |  |  |
| Sqrt Stem growth (cm^1^d^-1^) | Bacterial Trt | 0.056 | 0.972 |  |  |  |  |  |  |
|  | Init. Leaf SA | 2.074 | 0.15 |  |  |  |  |  |  |
|  | Bac + In. L. SA | 1.992 | 0.158 |  |  |  |  |  |  |
|  |  |  |  |  |  |  |  |  |  |
|  | Intercept |  |  | 0.244 | 0.087 | 2.808 | Exp. Run | 0.012 | 0.111 |
|  | GM30 |  |  | 0.082 | 0.082 | 0.994 | Residuals | 0.025 | 0.158 |
|  | GM41 |  |  | 0.079 | 0.079 | 0.992 |  |  |  |
|  | BT03 |  |  | 1.384 | 0.975 | 0.992 |  |  |  |
|  | Init. Leaf Num. |  |  | -0.003 | 0.082 | -0.038 |  |  |  |
|  |  |  |  |  |  |  |  |  |  |
| Log specific root length (cm^1^mg^-1^) | Bacterial Trt | 1.057 | 0.788 |  |  |  |  |  |  |
|  |  |  |  |  |  |  |  |  |  |
|  | Intercept |  |  | 2.498 | 0.760 | 3.291 | Exp. Run | 0.073 | 0.269 |
|  | GM30 |  |  | 1.7399 | 1.111 | 1.566 | Residuals | 0.208 | 0.456 |
|  | GM41 |  |  | 0.310 | 1.073 | 0.289 |  |  |  |
|  | BT03 |  |  | 0.506 | 1.111 | 0.455 |  |  |  |
| Log specific leaf area (cm^2^mg^-1^) |  |  |  |  |  |  |  |  |  |
|  | Bacterial Trt | 2.604 | 0.457 |  |  |  |  |  |  |
|  |  |  |  |  |  |  |  |  |  |
|  | Intercept |  |  | 0.219 | 0.037 | 5.996 | Exp. Run | 0.073 | 0.269 |
|  | GM30 |  |  | 0.001 | 0.047 | -0.024 | Residuals | 0.208 | 0.456 |
|  | GM41 |  |  | 0.038 | 0.046 | 0.84 |  |  |  |
|  | BT03 |  |  | -0.034 | 0.047 | -0.72 |  |  |  |
| Log Leaf Growth (cm^2^d^-1^) |  |  |  |  |  |  |  |  |  |
|  | Bacterial Trt | 9.211 | **0.01** |  |  |  |  |  |  |
|  | Init. Leaf SA | 0.313 | 0.575 |  |  |  |  |  |  |
|  | Bac + In. L. SA | 0.146 | 0.702 |  |  |  |  |  |  |
|  |  |  |  |  |  |  |  |  |  |
|  | Intercept |  |  | 0.235 | 0.088 | 2.658 | Exp. Run | 0.006 | 0.079 |
| Log Avg. Leaf Size  (cm^2^) | GM30 |  |  | 0.269 | 0.108 | **2.459** | Residuals | 0.044 | 0.21 |
|  | GM41 |  |  | 0.324 | 0.105 | **3.095** |  |  |  |
|  | BT03 |  |  | 0.121 | 0.109 | 1.149 |  |  |  |
|  |  |  |  |  |  |  |  |  |  |
|  | Bacterial Trt | 1.727 | 0.631 |  |  |  |  |  |  |
|  |  |  |  |  |  |  |  |  |  |
|  | Intercept |  |  | 0.722 | 0.26 | 2.778 | Exp. Run | 0.129 | 0.359 |
|  | GM30 |  |  | 0.211 | 0.221 | 0.953 | Residuals | 0.183 | 0.427 |
|  | GM41 |  |  | 0.143 | 0.214 | 0.67 |  |  |  |
|  | BT03 |  |  | -0.031 | 0.221 | -0.139 |  |  |  |
| Change in Leaf Size (cm^2^) |  |  |  |  |  |  |  |  |  |
|  | Bacterial Trt | 2.387 | 0.496 |  |  |  |  |  |  |
|  |  |  |  |  |  |  |  |  |  |
|  | Intercept |  |  | 2.184 | 0.706 | 3.093 | Exp. Run | 0.001 | 0.027 |
|  | GM30 |  |  | 0.909 | 0.671 | 1.354 | Residuals | 0.008 | 0.094 |
|  | GM41 |  |  | 0.076 | 0.648 | 0.118 |  |  |  |
|  | BT03 |  |  | 0.202 | 0.671 | 0.307 |  |  |  |
| Log carboxylase activity (slope of A/C_i_) |  |  |  |  |  |  |  |  |  |
|  | Bacterial Trt | 1.014 | 0.431 |  |  |  |  |  |  |
|  |  |  |  |  |  |  |  |  |  |
|  | Intercept |  |  | 0.158 | 0.023 | 6.927 |  |  |  |
|  | GM30 |  |  | 0.022 | 0.032 | 0.673 |  |  |  |
|  | GM41 |  |  | 0.051 | 0.03 | 1.687 |  |  |  |
|  | BT03 |  |  | 0.018 | 0.032 | 0.551 |  |  |  |
| A_sat_ |  |  |  |  |  |  |  |  |  |
|  | Bacterial Trt | 0.755 | 0.55 |  |  |  |  |  |  |
|  |  |  |  |  |  |  |  |  |  |
|  | Intercept |  |  | 4.092 | 2.415 | 1.694 |  |  |  |
|  | GM30 |  |  | 3.55 | 3.118 | 0.288 |  |  |  |
|  | GM41 |  |  | 2.597 | 2.958 | 0.878 |  |  |  |
|  | BT03 |  |  | 0.146 | 3.118 | 0.047 |  |  |  |
| A_max_ |  |  |  |  |  |  |  |  |  |
|  | Bacterial Trt | 1.978 | 0.188 |  |  |  |  |  |  |
|  |  |  |  |  |  |  |  |  |  |
|  | Intercept |  |  | 7.581 | 1.448 | 5.235 |  |  |  |
|  | GM30 |  |  | 4.454 | 2.048 | 2.175 |  |  |  |
|  | GM41 |  |  | 3.908 | 1.916 | 2.04 |  |  |  |
|  | BT03 |  |  | 2.315 | 2.048 | 1.13 |  |  |  |
|  |  |  |  |  |  |  |  |  |  |
|  |  |  |  |  |  |  |  |  |  |
|  |  |  |  |  |  |  |  |  |  |
|  |  |  |  |  |  |  |  |  |  |
|  |  |  |  |  |  |  |  |  |  |
|  |  |  |  |  |  |  |  |  |  |
